# Quantifying Mental Stress Using Cardiovascular Responses: A Scoping Review

**DOI:** 10.3390/s25144281

**Published:** 2025-07-09

**Authors:** Samira Ziyadidegan, Neda Sadeghi, Moein Razavi, Elaheh Baharlouei, Vahid Janfaza, Saber Kazeminasab, Homa Pesarakli, Amir Hossein Javid, Farzan Sasangohar

**Affiliations:** 1 Department of Industrial and Systems Engineering, Texas A&M University, College Station, TX 77840, USA; samiraziyadg@tamu.edu (S.Z.); moeinrazavi@tamu.edu (M.R.); amir.javid@tamu.edu (A.H.J.); 2 Department of Construction Science, Texas A&M University, College Station, TX 77840, USA; nsadeghi@tamu.edu; 3 Department of Computer Science, University of Houston, Houston, TX 77004, USA; ebaharlouei@uh.edu; 4 Department of Computer Science and Engineering, Texas A&M University, College Station, TX 77840, USA; vahidjanfaza@tamu.edu; 5 Harvard Medical School, Harvard University, Boston, MA 02115, USA; skazeminasabhashemabad@meei.harvard.edu; 6 Department of Architecture, Texas A&M University, College Station, TX 77840, USA; homa.pesarakli@tamu.edu

**Keywords:** mental stress, heart rate variability (HRV), physiological responses, quantitative methodologies, time series models

## Abstract

(1) Background: Physiological responses, such as heart rate and heart rate variability, have been increasingly utilized to monitor, detect, and predict mental stress. This review summarizes and synthesizes previous studies which analyzed the impact of mental stress on heart activity as well as mathematical, statistical, and visualization methods employed in such analyses. (2) Methods: A total of 119 articles were reviewed following the Systematic Reviews and Meta-Analyses extension for Scoping Reviews (PRISMA-ScR) guidelines. Non-English documents, studies not related to mental stress, and publications on machine learning techniques were excluded. Only peer-reviewed journals and conference proceedings were considered. (3) Results: The studies revealed that heart activities and behaviors changed during stressful events. The majority of the studies utilized descriptive statistical tests, including *t*-tests, analysis of variance (ANOVA), and correlation analysis, to assess the statistical significance between stress and non-stress events. However, most of them were performed in controlled laboratory settings. (4) Conclusions: Heart activity shows promise as an indicator for detecting stress events. This review highlights the application of time series techniques, such as autoregressive integrated moving average (ARIMA), detrended fluctuation analysis, and autocorrelation plots, to study heart rate rhythm or patterns associated with mental stress. These models analyze physiological data over time and may help in understanding acute and chronic cardiovascular responses to stress.

## 1. Introduction

According to a recent American Psychological Association (APA) survey, approximately one-third of the adult population in the United States feel overwhelmed due to daily stress [1]. The prevalence and impact of stress are even more pronounced in some special populations, such as college students. For example, a study conducted on 1472 medical students showed that 50.6% of the participants experienced mild and 37.0% moderate levels of stress [2]. Mental stress triggers a wide range of physical and psychological body responses [3]. A single stressful event or an ongoing circumstance stimulates a range of physiological responses that may negatively impact an individual’s health condition [4,5,6]. This includes an increased risk of cardiovascular diseases such as hypertension as well as mental disorders such as depression and anxiety [7,8,9,10].

The physiological responses to stress are associated with the Autonomic Nervous System (ANS) [11]. The ANS has two key components: the sympathetic and parasympathetic nervous systems (SNS and PNS) [12]. The PNS is responsible for regulating body functions during non-stress conditions, while the SNS prepares the body to respond to perceived threats or stressful events and initiate the “fight or flight” response [13]. Exposure to stress triggers the SNS to release hormones such as adrenaline and cortisol rapidly which causes a series of physiological changes in the body [14,15,16].

Significant changes in heart rate (HR) and heart rate variability (HRV) measurements have been documented during stressful events. In these studies, heart activity behaves more irregularly and less predictably to reflect the body’s responses to threats [14,17,18,19]. There is a clear trend towards the use of artificial intelligence (AI) techniques, particularly machine learning, to analyze heart rate activity data to assess stress [20,21,22,23]. One reason for such trends is the advances in sensor technology and the rise in cost-effective non-intrusive smart wearables equipped with HR and HRV sensors [22,24,25]. While AI-oriented techniques demonstrate potential for detection, such “black box” models are challenging to interpret and rarely help quantify or explain the physiological reactions to stress, particularly systemic changes in HR and HRV. In addition, time series data, particularly from off-the-shelf wearables, is considered “noisy” and warrants advanced analytical methods [15,16,17,26].

The goal of this research is to investigate the analytical methods used for HR and HRV data to better understand the heart’s reactions to mental stress, with the goal of developing non-intrusive stress monitoring tools. To our knowledge, two existing reviews have investigated similar analytical methods; however, they focused primarily on posttraumatic stress disorder (PTSD) [27] and anxiety [16]. This paper aims to document previous studies which used HR or HRV measurements to investigate mental stress. The review focuses on the use of mathematical, statistical, and visual methods, with emphasis on quantification techniques to study the effect of mental stress on heart rate behavior, rhythms, and patterns.

## 2. Materials and Methods

This review employs the scoping review approach to synthesize existing knowledge and identify the gaps. The process follows the Preferred Reporting Items for Systematic Reviews and Meta-Analyses Extension for Scoping Reviews (PRISMA-ScR) guidelines [28]. There is no scoping review protocol registered for this study.

### 2.1. Search Strategy

**Figure 1:**
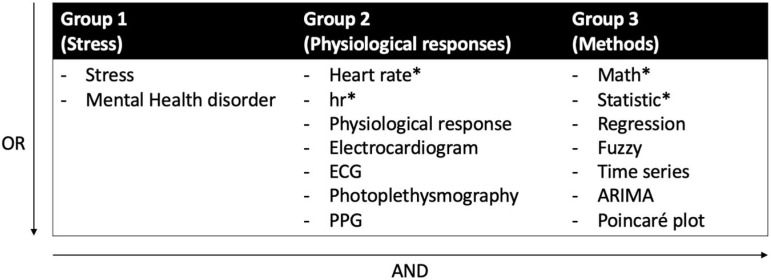
Study groups and search strategy.

Two databases, Compendex (Ei Village 2; Elsevier, Amsterdam, The Netherlands) and Google Scholar (Google, Inc., Mountain View, CA, USA), were searched in December 2023. Any peer-reviewed journal and conference papers that were related to the topic of analyzing cardiovascular responses to mental stress were selected using the following query and search terms (Figure 1): (Stress OR mental health disorder) AND (heart rate* OR hr* OR physiological response OR electrocardiogram OR ECG OR Photoplethysmography OR PPG) AND (math* OR statistic* OR Regression OR fuzzy OR time series OR ARIMA OR Poincaré plot).

### 2.2. Study Selection, Inclusion, and Exclusion Criteria

Documents not written in English were excluded. Publications focused on machine learning or deep learning techniques for detecting or analyzing mental health issues were also removed since a recent review [29] focused on such methods, and as discussed in the introduction, the main goal of this study was focusing on interpretable and quantification techniques which provide more clear insight into the relationships between different variables. Only peer-reviewed journal and conference proceeding papers were included.

## 3. Results

**Figure 2:**
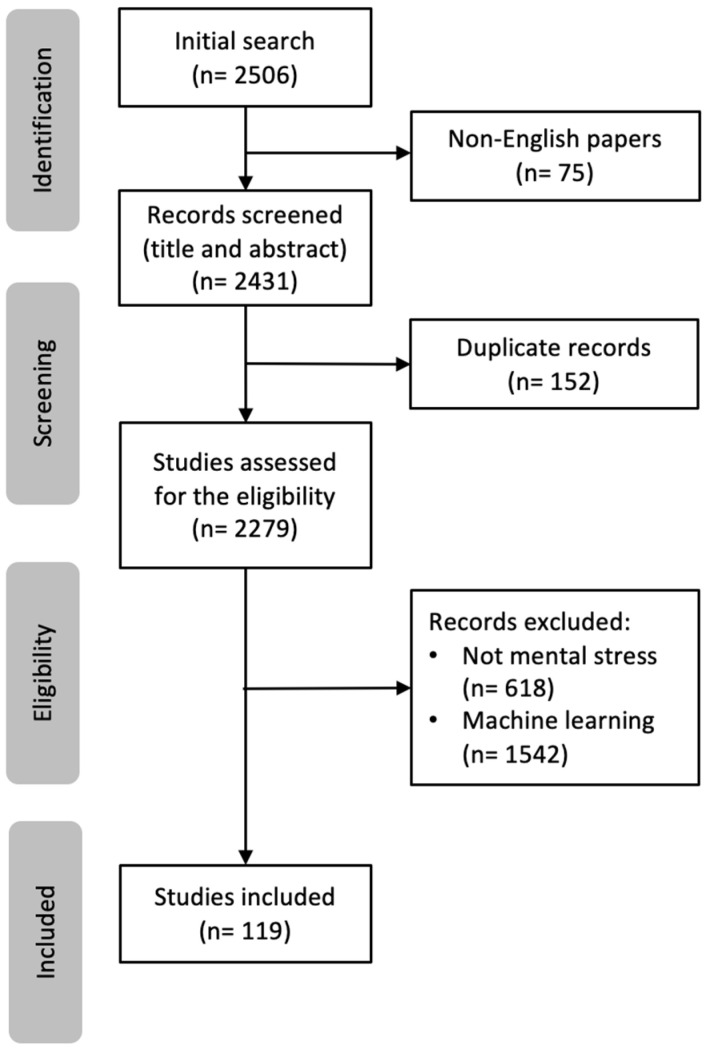
Diagram of article screening and selection.

The initial search resulted in 2506 articles (for studies published after 2010). After excluding non-English studies (n = 75) and duplicates (n = 152), the titles and abstracts of the articles were reviewed to assess their eligibility. This led to the elimination of an additional 2160 papers. Finally, 119 articles were selected to be used in this study. A summary of the reviewed papers is included in Table A1 in Appendix A. Figure 2 provides an overview of the review process. The reason for selecting the studies after 2010 is to focus more on the latest findings and methodologies researchers used and suggested in the stress domain.

### 3.1. Heart Activity Measurements and Their Responses to Stress

HR, also referred to as pulse rate or beats per minute (BPM), is the frequency of heart beats per minute. Studies provided information on how HR behaves in response to stress. HR increased during stressful events, as documented in [30,31,32]. A study also revealed that HR ranges more widely and exhibits more variability under stress conditions compared to non-stress ones [17]. In the following sections, stress-related changes in different HRV measurements have been categorized. HRV measured the variations in the interval between successive heartbeats over time. HRV can be numerically shown by time domain [14,33], frequency domain [14,33], and non-linear measurements [33,34,35].

### 3.2. Time-Domain HRV Measurements

Time-domain measurements measure the degree of variability in the inter-beat interval (IBI), which refers to the time between consecutive heartbeats (see Table A2 in Appendix B for a summary of parameters). These values can be represented in their base units or their natural logarithm to be more normally distributed [14,33]. Three time-domain measurements were used in the reviewed literature, namely, mean R-R interval, the standard deviation of NN intervals (SDNNs), and the root mean square of successive differences (RMSSD).

Mean R-R interval: this is the average time between heartbeats and has been observed to decrease during stressful events [11,36]. In a study on healthy volunteers, this measurement has been indicated as one of the most significant HRV time-domain measurements for distinguishing stressful events from non-stressful ones [37].

Standard deviation of NN intervals (SDNN): it is the standard deviation of heart inter-beat intervals [33]. Studies have revealed that SDNN has lower values in stress conditions compared to non-stress conditions [38,39].

The Root Mean Square of Successive Differences (RMSSD) is the root mean square of successive variations between heartbeats and has been found to decrease when an individual is under stress [39,40,41,42]. However, one study showed an increased RMSSD during stressful [43]. Additionally, de Vries concluded that RMSSD is not a significant predictor of stress [44].

### 3.3. Frequency-Domain HRV Measurements

Frequency-domain measurements calculate the variations between heart beats intervals in the frequency domain across three frequency bands: very-low-frequency (VLF), low-frequency (LF), and high-frequency (HF) bands [14,33] (see Table A3 in Appendix B for a summary of parameters). Three frequency-domain measurements were used in the reviewed literature: LF/HF ratio, Low Frequency (LF) power, and High Frequency (HF) power.

LF/HF ratio: the ratio of low- to high-frequency power, known as LF/HF, has been observed to increase during stressful events [39,43,45]. On the other hand, some studies indicated a decrease in the LF/HF ratio under stressful events [38,46,47]. Mae et al. underscored that the LF/HF ratio varies in response to mental stress [48].

Low-frequency (LF) power is the power of the low-frequency band measured between 0.04 and 0.15 Hz [33]. A few studies have observed that LF power increases during stressful events [32,49]. On the other hand, a study showed that LF power increases after a stress-relieving activity (Odissi dance) [43]. Similarly, one study found that LF power decreases significantly during stress compared to normal conditions [47].

High-frequency (HF) power represents the power of the high-frequency band (0.15 to 0.4 Hz) [33]. In multiple studies, HF power tends to decrease in response to stressful events [39,47]. Conversely, Chalmers et al. reported a significant rise in HF power during stressful events [32].

### 3.4. Non-Linear HRV Measurements

To study and quantify heart behaviors during stressful events, several techniques have been suggested and applied to the HRV time-domain or frequency-domain measurements (see Table A4 in Appendix B for a summary of parameters). HRV non-linear measurements are extracted from these models [33,34,35]. Three parameters were used in our reviewed literature, which are sample entropy and those extracted from detrended fluctuation analysis (DFA) and a Poincaré plot.

Sample entropy: Sample entropy is a metric used to assess the complexity and regularity of physiological time series data [33,50]. Castaldo et al. claimed that sample entropy decreased during stressful events [11].

Detrended fluctuation analysis (DFA): Fluctuation slopes, both short-term and long-term, show distinct patterns during stressful events (see Section 3.5.7 for more information about DFA and parameters) [33,51]. Castaldo et al. noted an increase in the slope of short-term fluctuations which implies an increase in short-term heart variabilities [11]. Conversely, they observed a decrease in the slope of long-term fluctuations which implies a decrease in long-term heart variabilities. Indeed, the value of the scaling component in DFA demonstrated significant differences between stress and non-stress events [52].

Poincaré plot [53]: Ramteke and Thool [42] found that a high level of stress is associated with a smaller standard deviation along both the perpendicular axis (SD1) and the parallel axis (SD2) in a Poincaré plot (see Section 3.5.5 for more information about Poincaré plot and parameters).

### 3.5. Analysis Methods

In this section, mathematical, statistical, and visualization techniques which have been used in the literature are studied. These techniques were used to understand and quantify heart activities during stressful events and to compare their behaviors with non-stress events.

**Figure 3:**
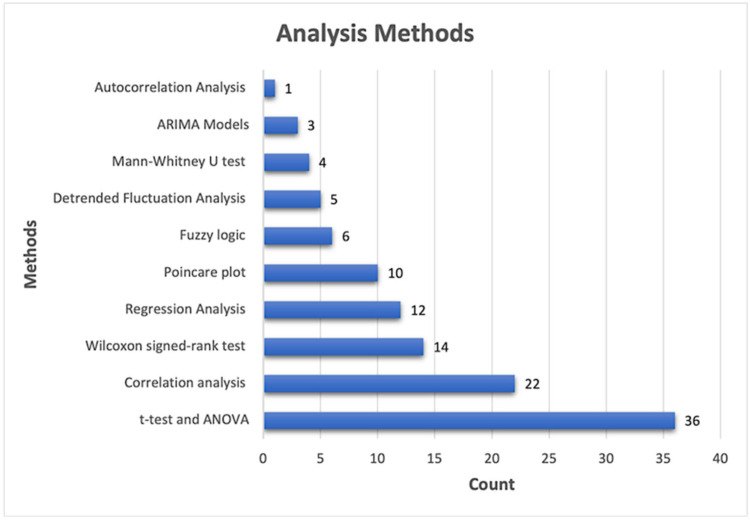
A summary of the number of the quantitative methods used in this study.

Figure 3 shows a summary of the frequency of the models used in the reviewed literature. The *t*-test and ANOVA are the most frequently employed statistical methods (n = 36), followed by correlation analysis (n = 22), Wilcoxon signed-rank test (n = 14), and regression analysis (n = 12). Other methods used are the Poincare plot (n = 10), fuzzy logic methods (n = 6), detrended fluctuation analysis (n = 5), and the Mann–Whitney U test (n = 4). ARIMA models (n = 3) and autocorrelation analysis (n = 1) are the least-utilized methods.

#### 3.5.1. *t*-Test and ANOVA (n = 36)

The Student’s *t*-test is utilized to compare the mean values between two groups, whereas the analysis of variance (ANOVA) is utilized when there are three or more groups. Studies used *t*-test [32,37,38,39,43,49,54,55,56,57,58,59,60,61,62,63,64,65,66,67,68,69,70,71,72,73,74,75,76,77], and ANOVA [40,47,49,67,75,78,79,80,81] to check if the HR or HRV parameters are meaningfully different between stress and non-stress events or between different levels of stress (low, medium, and high).

#### 3.5.2. Correlation Analysis (n = 22)

Pearson correlation [17,32,36,61,62,77,82,83,84,85,86,87,88,89,90], Spearman correlation [36,72,91,92,93], complex correlation [94], P_k_ measurement [95], or cross-correlation [95] were used to find the relationships between different HRV measurements, the stress scores or levels extracted from questionnaires, and participants’ demographic data (e.g., gender or educational background).

#### 3.5.3. Wilcoxon Signed-Rank Test (n = 14)

The Wilcoxon signed-rank test is a non-parametric statistical test which is employed to compare the values from dependent data when they are not normalized [96]. This test can be used to identify the significant differences between heart activities (e.g., HRV measurements) or other measurements of stress events (e.g., self-reported stress scores) within the same participants in different studies [11,38,46,60,76,91,92,93,97,98,99,100,101,102]. For example, in the study conducted by Castaldo et al., since multiple HRV measurements were not normally distributed, this test was applied to study if HRV measurements significantly changed during acute stressful events [11].

#### 3.5.4. Regression Analysis (n = 12)

Regression analysis is used to determine the magnitude and direction of associations between dependent and independent variables [103]. Regression analysis has been utilized in the literature to detect stress levels using a linear regression model [104] and a non-linear regression model [66]. In other studies, significant predictors of stress were extracted using logistic regression [59,65] and the stepwise regression method [105]. Also, regression methods were used to evaluate the correlation between different parameters [17,82,83,89,101,106]. For example, Sadeghi et al. [17] utilized a linear regression model to study the impact of several factors, including hyperarousal events, demographic data, medical treatments, and lifestyle factors, on resting HR of veterans with PTSD.

#### 3.5.5. Poincare Plot (n = 10)

A Poincaré plot is a geometrical representation used to assess the correlation between two subsequent data in a time series dataset [107]. In the stress domain, several studies plot the R-R interval (RR_n_) versus the consecutive R-R interval (RR_n+1_) to analyze the changes during stressful events. To do so, an ellipse is fitted to the data which is oriented to the identity line (y = x line). The SD1 width and SD2 length (see Table A4 in Appendix B for more information) correspond to the short- and long-term variability of the R-R intervals, respectively. It is hypothesized that SD1 is an indicator of parasympathetic activity, while SD2 and the SD1/SD2 ratio are indicators of sympathetic activity [108].

Previous studies determined that the Poincaré plot’s elliptical shape changed and became narrower and more confined (shorter SD1 and SD2) under stressful events and became wider under more relaxed events [42,53,60,64,81,100,108,109,110,111]. During stressful events, since sympathetic activity increases due to an inverse relationship between SD2 and sympathetic activity, SD2 length becomes shorter. Similarly, due to the decrease in parasympathetic activity, SD1 width becomes shorter as well [108]. The same study showed that the SD1/SD2 ratio exhibited an inverse trend and increased significantly during stressful events [108]. Conversely, Pereira et al. [100] employed the Poincaré plot to study the effects of transitioning from normal to stressful conditions and revealed a significant decrease in both SD1 and SD2 values.

#### 3.5.6. Fuzzy Logic (n = 6)

The phrase “fuzzy” refers to unclear information [112]. Fuzzy logic is a method of calculating considering “degrees of truth” (between 0 and 1), as opposed to binary “true or false” Boolean logic (0 and 1) [112,113]. This logic is useful to address the vagueness of situations, particularly when the distinctions between categories are not clear.

Fuzzy logic using heart activity as input variables has been used to define and utilize fuzzy logic rules to identify different stress levels, e.g., low, medium, and high [114] or relaxed and stressed situations [115]. Sul et al. [116] used fuzzy logic to predict the degree of stress as a value between 0 and 1. Kumar et al. [117] used a stochastic fuzzy method to estimate the stress level for individuals using R-R intervals. The suggested approach was used in a mobile telemedical application to consider the participants’ uncertainties. Airij et al. [55] suggested a fuzzy logic with eight rules to detect stress using physiological responses, including HR data. The results revealed that fuzzy logic has higher accuracy (96.19%) than machine learning models. Chen et al. [118] developed a portable device that fuses multiple sensors, utilized Gaussian membership functions, and suggested 81 fuzzy rules to assess the accumulative stress levels of individuals.

#### 3.5.7. Detrended Fluctuation Analysis (n = 5)

Detrended fluctuation analysis (DFA) [119,120] identifies long-range correlations and self-affinity in time series data. DFA imposes less strict assumptions on the stationarity of the time series which makes it a more flexible option for time series data analysis, e.g., heart activity data [120,121,122]. The fractal-like pattern of HRV determines that various physiological behaviors occur over different time periods. DFA can discover these patterns and any changes which occur during stressful events. This can be achieved by analyzing DFA-extracted parameters of HRV in stress studies. One study evaluated the DFA scaling exponent of the R-R interval plot to detect stress [52]. Other studies focused on long- and short-term fluctuations in R-R interval time series data [11,17,60,100]. All of these studies have shown significant changes in DFA-extracted parameters during stressful events.

#### 3.5.8. Mann–Whitney U Test (n = 5)

Mann–Whitney U test is another non-parametric statistical test which is frequently utilized to compare the mean values from independent data when the data is not normal [123]. This test has been used to compare the measurements of distinct groups of stress and non-stress in terms of creative performance [38,39] or to compare the median of ultra-short HRV indices and cardiac cycle parameters between non-stress and stress events [99]. Jo et al. [46] applied the Mann–Whitney U test and claimed that the stressed group exhibited a significantly different LF/HF ratio compared to the non-stressed group. Salahuddin and Kim [124] utilized this test to compare the trends of the HRV measurements between non-stress and stressed conditions.

#### 3.5.9. ARIMA (Autoregressive Integrated Moving Average) Models (n = 3)

ARIMA [125,126,127] models have been used to examine time series data by detecting correlations over time to recognize trends and predict based on the lagged values [86]. A study conducted by Choi and Gutierrez-Osuna [86] proposed a novel approach for detecting mental stress using HRV measurements and breathing data by using an autoregressive moving average with exogenous inputs (ARMAX) model. They analyzed the influence of breathing on heart rate variability measurements to discriminate between mental stress and relaxation conditions. Autoregressive models can also be used in the data pre-processing analysis and extracting HRV measurements to be studied in the stress domain [11]. de Vries et al. [44] used a vector autoregressive model and showed that RMSSD is not a significant predictor of stress.

#### 3.5.10. Autocorrelation Analysis (n = 1)

Autocorrelation analysis determines how data in a time series are associated with their lagged values [128]. One study used autocorrelation plots of HR or HRV measurements to evaluate the trends and patterns of these physiological parameters during stressful events and to compare their distinct pattern to normal events [17].

#### 3.5.11. Other Methods (n = 6)

There are several other quantitative methods which were employed in the literature. Shao, Zhou, Wang et al. [129] used the area under the HR waveform to analyze the HR trends before, during and after special training for pilots (stressful events). Sarkar et al. [130] provided a 3D phase space plot in a spherical coordinate system that effectively differentiates between relaxed and stressed states. Garcia-Mancilla & Gonzalez [31] used descriptive analysis to find the relationships between stress, HR, and other physiological responses. Raj et al. [45] also employed descriptive analysis to study changes and patterns in HRV measurements during stressful events. Hooker et al. [131] utilized chi-squared test and covariance analysis to investigate the cardiovascular effects of receiving texts from a significant other while under stress. Cubillos-Calvachi et al. [132] compared various conditions and cardiac-related criteria in diagnostic tests using descriptive analysis to evaluate the impact of stressful situations on students.

### 3.6. Experimental Settings

Most studies were conducted in a lab/controlled environment where stress was induced and HR and HRV reactions to such stressful events were measured. Various stress-inducing methods and assessment tools were employed, as follows:

Social stressors included situations with induced social anxiety and pressure. Methods used to induce social stress included negative performance feedback [38], Trier Social Stress Test [32,56,65,98,111,133,134], public speaking/speech task/oral presentation/oral exam [37,45,57,87,91,92,102,131], semi-structured social competence interview [66], and Sing-a-Song Stress Test (SSST) [40,68].

Physical/mental stressors included tasks and stimuli that make the body and mind stressed. They included physical/mental stimuli [118,135], the threat of not painful shock [38], inducing a sense of urgency with a countdown screen [69], noise exposure (noise test) [40], special training/flights for pilots [47,129], emergency events during flights [85], supine and tilt [87], exploring a virtual environment [63], and playing loud one-second white noise [71].

Cognitive stressors included cognitive and mental-challenging tasks, such as arithmetic operations/tests/game [53,54,55,60,77,79,83,84,89,95,99,101,109,131], n-back test [36,40], task switching [49], neuropsychological d2 test of attention [36], Trail Making Test (TMT) [136], color Stroop test [11,37,49,58,64,87,106,109], the Montreal Imaging Stress Task (MIST) [61,69], memory search [87], dual tracking tasks [87], mirror tracing [87], minesweeper game [46], sudoku puzzle [130], chess game [130], coin stacking task [116], prisoners’ red and blue cap problem [130], exams [42,67,81,88,132,137], 3D image manipulation and pattern finding [138], video games [97,110], target tracking and memory search tasks [86], solving 3D puzzles [114,115,139], and driving simulators [105].

Assessment tools included tools which measure stress levels or scores, such as the Montreal Imaging Stress Task (MIST) [37,140], the State/Trait Anxiety Inventory (STAI) [59,62,75,82,88,98,100], the NASA Task Load Index (NASA-TLX) [62], the Perceived Stress Scale (PSS) [46,62,104,118], the Mini-Social Phobia Inventory (Mini-SPIN) self-report questionnaire [80], the Liebowitz Social Anxiety Scale (LSAS) questionnaire [80], the Depression Anxiety Stress Scale (DASS) [32,43,138], the Daily Stressor and Supportive Events (DSSQ) test [78], the Technostress Creators questionnaire [141], the Visual Analogue Scale (VAS) [93], self-report by mobile/watch application [17], the Shirom–Melamed Burnout Questionnaire (SMBQ) [82], customized questionnaires [55,63,72,93,94], the Hamilton Depression Rating Scale (HDRS) [70,76], the Social Phobia Inventory (SPIN) [73], the Diagnostic and Statistical Manual of Mental Disorders (DSM-5) [70], and the Four-Dimensional Symptom Questionnaire (4DSQ) [44].

On the other hand, fewer naturalistic studies were conducted, where stress was studied in a real-world or naturalistic environment [17,31,40,47,57,72,73,85,89,93,104,132,141,142]. In such environments, studies used wearable sensors, e.g., smart watches [17] or chest sensors [141], as opposed to more complex and non-intrusive Electrocardiogram (ECG) or Photoplethysmogram (PPG) sensors, e.g., electrodes [38,82,83], utilized in lab settings.

## 4. Discussion

### 4.1. Heart Rate Metrics: Promising Indicators for Mental Stress Detection

While this paper focused on reviewing the methods used to analyze heart rate activity data, this review revealed that, in line with previous findings [14,143], HR and HRV measurements showed promise for detecting and predicting stressful events. During such events, the balance between sympathetic and parasympathetic nervous system activities is altered, which affects heart behaviors. While it is well-documented that during stressful events, the Mean R-R interval decreases, and heart rate experiences increased variability compared to non-stress/relaxed conditions, this review showed some discrepancies. For example, while some studies report an increase in LF/HF ratios during stressful events [38,45], others claim that this ratio decreased after stressful events [36,47]. The highlighting factors for these discrepancies are different participants’ characteristics (e.g., athletic students vs. employees of a medical center, or different age groups), the method types they utilized (e.g., correlation analysis vs. *t*-test), environmental settings (lab vs. naturalistic), and different stressor types (solving a puzzle vs. singing a song). Despite such discrepancies, this review confirms that there is sufficient evidence of significant changes in heart activities during the pre- and post-stress periods. These findings highlight the potential for investigating heart rate rhythm and patterns for stress detection or monitoring.

### 4.2. Statistical Analysis Methods or Models for Heart-Related Data

This review revealed that the majority of the studies utilized statistical tests, particularly *t*-tests, ANOVA, and correlation analysis, to assess the differences between stress and normal events. These findings highlighted the utility of these methods in the cardiovascular-based stress analysis domain to provide a robust framework for analyzing and interpreting data [144]. However, most analysis models used averaged values (e.g., stress vs. non-stress group heart rates), which may overlook the individual variations and long-term variations. Additionally, one of the main limitations of these methods is the ability to address the inherent noise in the data due to the effect of other cognitive or physical conditions, such as exercise, which may explain the discrepancies in reported findings.

### 4.3. Utility of Time Series Analysis Methods

HR and HRV are inherently temporal physiological body responses, and it is important to utilize time series techniques to discover the temporal changes in response to stress. Therefore, in addition to statistical tests, this review highlighted the importance of the application of time series analysis methods and models in the heart activity-based investigation of mental stress. This is in line with a few studies which underscore the importance of studying time series analysis in the stress domain [16,27]. Time series analysis, including ARIMA, DFA, and autocorrelation plots, provides a more in-depth understanding of temporal changes and patterns of heart variations during stressful events [128]. Moreover, because time series analysis can show correlations between the current and lagged values of HR and HRV measurements, e.g., in correlation plots, this method can be used to assess both the immediate and delayed heart responses to stress [128,145]. For example, using autocorrelation and DFA techniques can help in analyzing short-term and long-term effects of stress on heart activity. This idea has been studied by Ziyadidegan and Sasangohar, and the ensuing results will be presented in future publications.

### 4.4. Experimental Settings: Naturalistic or Laboratory

A few studies collected data when the participants were under stress in their real-world stress activities [21,72]. On the other hand, most of the reviewed studies collected data in lab settings where different types of stress-inducing tasks or tests, including social, physical/mental, and cognitive stressors, were used [37,38]. Although lab studies provide the benefit of a controlled environment, it is not clear if the stress induced in such settings would be generalized to stress in the real world. In addition, the data collected in such settings might suffer from other biases and new stressors, such as the existence of other participants or examiners, which affect their validity. Future work should also prioritize utilizing data collected using non-intrusive wearable devices in naturalistic environments to collect data continuously during participants’ daily life activities. This approach would not only improve the practical relevance of the resulting solutions, but may also unveil several contextual barriers to inform the design of future wearable systems.

### 4.5. Gaps and Limitations

This review has several noteworthy limitations. First, although this paper reviewed the area of stress quantification and analysis using heart activity data, it might not cover all aspects of this field comprehensively. A systematic review might be needed to ensure all papers from various fields of study are included. Second, the reviewed studies utilized different methods, experimental designs, and participant characteristics (see Table A1 in Appendix A for a summary). This may be the reason for the various conflicting results reported in this study. Future work is needed to evaluate the study designs to confirm the generalizability and reliability of the results. It is also suggested to use a meta-analysis technique that utilizes different statistical and quantification techniques to combine/assess results from literature with different designs and participant characteristics to determine the overall impact of stress on heart activity [146]. Finally, most studies did not include information about the personal characteristics of participants, e.g., their resting heart rate or whether they were athletes. These characteristics might affect the reliability and accuracy of their findings.

## 5. Conclusions

The growing interest in utilizing off-the-shelf and non-intrusive heart rate sensors in the stress domain motivated the need to comprehensively review methods to analyze data from such sensors. The review showed that while various methods have shown promise in analyzing heart activity data, several important limitations remain. Most importantly, the majority of the studies were conducted in controlled lab settings, which allowed the examiner to gather data in a more controlled environment. However, they might result in potentially unrealistic stress induction and the development of new biases. It is suggested to use non-intrusive wearable data collection devices to collect data within participants’ real-world activities. Moreover, many of these studies employed descriptive statistics or threshold-based methods to study stress and compared heart activity with non-stress conditions. However, such models cannot differentiate between stress-induced responses and those caused by other activities, e.g., exercising. Advanced methods such as trend analyses and studies in naturalistic settings are warranted to better understand the effects of stress (particularly in the long term) on heart rate rhythm and pattern changes. One idea is to use advanced time series techniques to clearly learn the temporal changes in heart activities in response to stress.

## Data Availability

Data are contained within the article and Appendix A and Appendix B.

## Appendix A

The Summary of articles included in the review is provided here.

**Table A1:** Summary of articles included in the synthesis sorted by publication date.

Article	Year	Population Type	Method Name	Environmental Setting	Stressors
[86]	2010	Human participants (N = 4)	- Pearson correlation - ARIMA	Lab	Target tracking and memory search tasks
[95]	2010	N = 1	Cross-correlation	Lab	Mental calculation
[38]	2011	Healthy undergraduate students (N = 37)	- *t*-test - Wilcoxon signed-rank test - Mann–Whitney U test	Lab	- Negative performance feedback - Threat of not painful shock
[94]	2011	Male firefighters (N = 25)	Correlation analysis	Naturalistic	Self-designed questionnaire
[49]	2011	Freshman undergraduate students (N = 48)	- *t*-test - ANOVA	Lab	- Task switching - Color Stroop test
[102]	2011	Human participants (N = 28)	Wilcoxon signed-rank test	Lab	Oral answering
[87]	2012	Human participants (N = 19)	Pearson correlation	Lab	- Memory search - Dual tracking task - Mirror tracing - Color Stroop test - Public speech
[117]	2012	Human participants (N = 50)	Fuzzy logic	Lab	N/A
[46]	2012	Healthy subjects (N = 32)	- Wilcoxon signed-rank test - Mann–Whitney U test	Lab	- Perceived stress scale (PSS) - Minesweeper game
[106]	2012	Healthy participants (N = 21)	Regression analysis	Lab	- Color Stroop test
[85]	2013	Firefighter (N = 4)	Pearson correlation	Naturalistic	Emergency events during flights
[73]	2014	Students (N = 8)	*t*-test	Lab	Social Phobia Inventory (SPIN)
[115]	2014	Healthy volunteers	Fuzzy logic	Lab	Solving a 3D puzzle
[118]	2014	Males (N = 17)	Fuzzy logic	Naturalistic	- The Perceived Stress Scale (PSS) - Physical/mental stimuli
[11]	2015	healthy subjects (N = 42)	- Wilcoxon signed-rank test - ARIMA - DFA	Lab	Color Stroop test
[124]	2015	Healthy subjects (N = 11)	Mann–Whitney Wilcoxon test	Lab	Stroop color word test
[64]	2015	Public safety worker (N = 39)	- Poincaré plot - *t*-test	Lab	Color Stroop test
[111]	2015	Healthy students	Poincaré plot	Lab	Trier Social Stress Test
[80]	2015	Undergraduate students (N = 73)	ANOVA	Lab	- The Mini-Social Phobia Inventory (Mini-SPIN) self-report questionnaire - The Mini-Social Phobia Inventory (Mini-SPIN) self-report questionnaire
[31]	2015	Human participants (N = 1)	Descriptive Visualization analysis	Naturalistic	Keep a diary of stressful events
[54]	2015	Healthy engineering university students (N = 30)	*t*-test	Lab	Mental Arithmetic Task (MAT)
[77]	2016	Male participants (N = 34)	- *t*-test - Pearson correlation	Lab	Arithmetic Operations/tests/game
[114]	2016	Human participants (N = 68)	Fuzzy Logic	Lab	3D Puzzle
[68]	2016	Human participants (N = 8)	*t*-test	Lab	Sing-a-Song Stress Test (SSST)
[98]	2016	Healthy volunteers (N = 46)	Wilcoxon signed-rank test	Lab	- Trier Social Stress Test - State/Trait Anxiety Inventory (STAI)
[137]	2016	Students (N = 13)	Data visualization	Lab	Multiple-choice questions exam
[53]	2017	Human participants (N = 9)	- Complex correlation - Poincaré plot	Lab	Arithmetic operations/tests/game
[83]	2017	Human participants (N = 65)	- Pearson correlation - Regression Analysis	Lab	Arithmetic operations/tests/game
[78]	2017	Twenty-two participants (N = 22)	ANOVA	Lab	Daily stressor and supportive events (DSSQ) test
[100]	2017	Human participants (N = 14)	- Wilcoxon signed-rank test - Poincaré plot - DFA	Lab	State/Trait Anxiety Inventory (STAI)
[134]	2017	Students (N = 46)	Friedman test	Lab	Trier Social Stress Test
[42]	2017	students (N = 30)	Poincaré plot	Lab	Exam
[109]	2017	Human participants (N = 7)	Poincaré plot	Lab	- Stroop color–word test (CWT) - Mental arithmetic test
[133]	2018	Human participants (N = 26)	Regression approach based on separability maximization	Lab	Trier Social Stress Test
[104]	2018	Healthy people (N = 8)	Linear regression model	Naturalistic	The Perceived Stress Scale (PSS)
[67]	2018	University students (N = 33)	- *t*-test - ANOVA	Lab	Exam
[82]	2018	Human participants (N = 47)	- Pearson correlation - Regression Analysis	Lab	- State/Trait Anxiety Inventory (STAI) - Shirom-Melamed Burnout questionnaire (SMBQ)
[55]	2018	Human participants (N = 35)	- *t*-test - Fuzzy logic	Lab	- Designed Questionnaire - Arithmetic Operations/tests/game
[97]	2018	Healthy subjects (N = 69)	Wilcoxon signed-rank test	Lab	Video game
[70]	2018	MDD patients and control group (N = 11 each)	*t*-test	Lab	- Diagnostic and Statistical Manual of Mental Disorders (DSM-5) - Hamilton Rating Scale for Depression
[131]	2018	Female participants(N = 75)	- Chi-squared test - Covariance analysis	Lab	Speech and math task
[56]	2018	healthy students (N = 40)	*t*-test	Lab	Trier Social Stress Test
[93]	2018	Male firefighters (N = 6)	- Spearman correlation - Wilcoxon signed-rank test	Naturalistic	- The Visual Analogue Scale (VAS) - Designed questionnaire
[81]	2018	Healthy students (N = 90)	- Poincaré plot - ANOVA	Lab	Exam
[66]	2018	Young adults (N = 98) Married couples (N = 60)	*t*-test Non-linear regression analysis	Lab	Semi-structured Social Competence Interview
[47]	2019	Male pilots (N = 11)	ANOVA	Naturalistic	Special training/flight
[129]	2019	Pilots	A quantitative analysis method using the area of HR waveform	Naturalistic	Special training/flight
[88]	2019	Students (N = 40)	Pearson correlation	Lab	State/Trait Anxiety Inventory (STAI)
[99]	2019	Healthy participants (N = 16)	- Wilcoxon signed-rank test - Mann–Whitney U test	Lab	Arithmetic tests
[101]	2019	young, healthy participants (N = 76)	- Wilcoxon signed-rank test - Regression Analysis	Lab	Mental arithmetic test
[79]	2019	Healthy participants (N = 6)	ANOVA	Lab	Mental arithmetic test
[58]	2019	Young, healthy participants (N = 10)	*t*-test	Lab	Color Stroop test
[57]	2019	Human participants (N = 16)	*t*-test	Naturalistic	Speech task
[92]	2019	Students (N = 42)	Spearman correlation Wilcoxon signed-rank test	Naturalistic	Oral Exam
[91]	2019	Healthy participants (N = 42)	- Spearman correlation - Wilcoxon signed-rank test	Naturalistic	Oral Exam
[139]	2020	Engineering student (N = 42)	Fuzzy logic	Lab	Solving 3D puzzle
[132]	2020	Students (N = 50)	Descriptive statistical analysis	Naturalistic	Exam
[105]	2020	Healthy male (N = 5)	Stepwise regression method	Lab	Driving Simulator
[37]	2020	Healthy volunteers (N = 36)	*t*-test	Lab	- Speech task - The Montreal Imaging Stress Task (MIST) - Color Stroop test
[62]	2020	Moderately stressed participants (N = 13)	- *t*-test - Pearson correlation	Lab and naturalistic	- State/Trait Anxiety Inventory (STAI) - The NASA Task Load Index (NASA-TLX) - The Perceived Stress Scale (PSS)
[74]	2020	Patient (N = 1)	*t*-test	Lab	Rehabilitation sessions
[135]	2020	volunteers and colleagues (N = 6)	Descriptive statistical analysis	Lab	Physical/mental stimuli
[72]	2020	Male firefighters (N = 26)	- *t*-test - Spearman correlation	Naturalistic	Designed questionnaire
[76]	2020	Healthy and depressed patients (N = 82, 36 healthy volunteers, 46 patients with moderate depression)	- *t*-test - Wilcoxon signed-rank test	Lab	Hamilton Depression Rating Scale (HDRS)
[89]	2020	High-school student (N = 139)	- Pearson correlation - Regression Analysis	Naturalistic	Math test
[84]	2020	Human participants (N = 29)	Pearson Correlation	Lab	Arithmetic operations/tests/game
[110]	2020	Human participants (N = 14)	- Poincaré plot	Lab	Virtual reality video game
[60]	2020	Healthy undergraduate students (N = 20)	- *t*-test - Wilcoxon signed-rank test - Poincaré plot - DFA	Lab	Arithmetic operations/tests/game
[63]	2020	Human participants (N = 27)	- *t*-test	Lab	- Exploring a virtual environment - Self-designed questionnaires
[130]	2020	Human participants (N = 6)	3D phase space plot	Lab	- Sudoku puzzle - Chess game - Prisoners’ red and blue cap problem
[61]	2020	Human participants (N = 18)	- *t*-test - Pearson correlation	Lab	The Montreal Imaging Stress Task (MIST)
[65]	2021	Graduate students (N = 17)	- *t*-test - logistic regression	Lab	Trier Social Stress Test
[36]	2021	Healthy subjects (N = 10)	- Pearson correlation - Spearman correlation	Lab	- N-back test - Neuropsychological d2 Test of Attention
[69]	2021	Undergraduates and postgraduates (N = 16)	*t*-test	Lab	- Giving a sense of urgency with countdown screen - The Montreal Imaging Stress Task (MIST)
[59]	2021	Human participants (N = 48)	- *t*-test - Logistic regression	Lab	State/Trait Anxiety Inventory (STAI)
[75]	2021	Elderly people (N = 7)	- *t*-test - ANOVA	Lab	State/Trait Anxiety Inventory (STAI)
[71]	2021	Human participants (N = 5)	*t*-test	Lab	Playing loud one-second white noise
[45]	2021	Employees of a medical center (N = 44)	Descriptive statistical analysis	Lab	Oral presentation
[17]	2021	Veterans with PTSD (N = 99)	- Pearson correlation - Autocorrelation analysis - DFA - Regression Analysis	Naturalistic	Self-report by mobile/watch application
[90]	2022	Human participants (N = 2)	Correlation analysis	Lab	N/A
[40]	2022	Human participants (N = 24)	ANOVA	Naturalistic& Lab	- Sing-a-Song Stress Test (SSST) - Noise Exposure (Noise Test) - N-back test
[32]	2022	MD students and general people (N = 60, 30 MD students, 30 general people)	- *t*-test - Pearson correlation	Lab	- Depression Anxiety Stress Scale (DASS) - Trier Social Stress Test
[43]	2023	Students (N = 103)	- *t*-test	Lab	Depression Anxiety Stress Scale (DASS)
[44]	2023	Police officers (N = 8)	Vector Autoregression (VAR) modeling	Naturalistic	The Four-Dimensional Symptom Questionnaire (4DSQ)
[39]	2023	Athlete students (N = 41)	- *t*-test - Mann–Whitney U test	Lab	Arithmetic operations/tests/game

## Appendix B

The Summary of heart activity measurements included in the review is provided here.

**Table A2:** HRV time-domain features; source [14,33].

Parameter	Unit	Description
SDNN	ms	Standard deviation of NN intervals
SDRR	ms	Standard deviation of RR intervals
SDSD	ms	Standard deviation of differences between adjacent NN intervals
SDANN	ms	Standard deviation of the average NN intervals for each 5 min segment of a 24 h HRV recording
SDNN index (SDNNI)	ms	Mean of the standard deviations of all the NN intervals for each 5 min segment of a 24 h HRV recording
NN50 count		The total count of adjacent NN interval pairs that are more than 50 ms apart over the whole recording.
pNN50	%	Percentage of successive RR intervals that differ by more than 50 ms
HR_max_–HR_min_	bpm	Average difference between the highest and lowest heart rates during each respiratory cycle
RMSSD	ms	Root mean square of successive RR interval differences
HRV triangular index	-	The integral of the density of the RR interval histogram divided by its height
TINN	ms	Baseline width of the RR interval histogram
SDANN	ms	Standard deviation of the average NN intervals for each 5 min segment of a 24 h HRV recording

**Table A3:** HRV frequency-domain features; source [14,33].

Parameter	Unit	Description
ULF power	ms^2^	Absolute power of the ultra-low-frequency band (≤0.003 Hz)
VLF power	ms^2^	Absolute power of the very-low-frequency band (0.0033–0.04 Hz)
LF peak	Hz	Peak frequency of the low-frequency band (0.04–0.15 Hz)
LF power	ms^2^	Absolute power of the low-frequency band (0.04–0.15 Hz)
LF power	nu	Relative power of the low-frequency band (0.04–0.15 Hz) in normal units
LF power	%	Relative power of the low-frequency band (0.04–0.15 Hz)
HF peak	Hz	Peak frequency of the high-frequency band (0.15–0.4 Hz)
HF power	ms^2^	Absolute power of the high-frequency band (0.15–0.4 Hz)
HF power	nu	Relative power of the high-frequency band (0.15–0.4 Hz) in normal units
HF power	%	Relative power of the high-frequency band (0.15–0.4 Hz)
LF/HF	%	Ratio of LF-to-HF power

**Table A4:** HRV non-linear features, Source [14,33].

Parameter	Unit	Description
S	ms	Area of the Poincaré plot ellipse, representing total HRV
SD1	ms	Poincaré plot standard deviation, perpendicular to the line of identity; indicator of parasympathetic activity
SD2	ms	Poincaré plot standard deviation, along the line of identity; indicator of sympathetic activity
SD1/SD2	%	Ratio of SD1 to SD2; an indicator of sympathetic activity
ApEn		Approximate entropy, which measures the regularity and complexity of a time series
SampEn		Sample entropy, which measures the regularity and complexity of a time series
DFA α1		Detrended fluctuation analysis parameter, which describes short-term fluctuations
DFA α2		Detrended fluctuation analysis parameter, which describes long-term fluctuations
DFA α		Detrended fluctuation analysis scaling component
